# The Relationship Between Grain Hardness, Dough Mixing Parameters and Bread-Making Quality in Winter Wheat

**DOI:** 10.3390/ijms13044186

**Published:** 2012-03-30

**Authors:** Bolesław P. Salmanowicz, Tadeusz Adamski, Maria Surma, Zygmunt Kaczmarek, Krystkowiak Karolina, Anetta Kuczyńska, Zofia Banaszak, Bogusława Ługowska, Małgorzata Majcher, Wiktor Obuchowski

**Affiliations:** 1 Institute of Plant Genetics, Polish Academy of Sciences, Strzeszyńska Str. 34, 60–479 Poznań, Poland; E-Mails: tada@igr.poznan.pl (T.A.); msur@igr.poznan.pl (M.S.); zkac@igr.poznan.pl (Z.K.); kkry@igr.poznan.pl (K.K.); akuc@igr.poznan.pl (A.K.); 2 DANKO Plant Breeding Ltd., Choryń, Poland; E-Mails: zofia.banaszak@danko.pl (Z.B.); boguslawa.lugowska@danko.pl (B.Ł.); 3 Poznań University of Life Sciences, Wojska Polskiego 31, 60-624 Poznań, Poland; E-Mails: majcherm@up.poznan.pl (M.M.); obuchows@up.poznan.pl (W.O.)

**Keywords:** wheat, kernel hardness, puroindoline alleles, protein content, mixing parameters

## Abstract

The influence of grain hardness, determined by using molecular markers and physical methods (near-infrared (NIR) technique and particle size index—PSI) on dough characteristics, which in turn were determined with the use of a farinograph and reomixer, as well as bread-making properties were studied. The material covered 24 winter wheat genotypes differing in grain hardness. The field experiment was conducted at standard and increased levels of nitrogen fertilization. Results of molecular analyses were in agreement with those obtained by the use of physical methods for soft-grained lines. Some lines classified as hard (by physical methods) appeared to have the wild-type *Pina* and *Pinb* alleles, similar to soft lines. Differences in dough and bread-making properties between lines classified as hard and soft on the basis of molecular data appeared to be of less significance than the differences between lines classified as hard and soft on the basis of physical analyses of grain texture. Values of relative grain hardness at the increased nitrogen fertilization level were significantly higher. At both fertilization levels the NIR parameter determining grain hardness was significantly positively correlated with the wet gluten and sedimentation values, with most of the rheological parameters and bread yield. Values of this parameter correlated with quality characteristics in a higher degree than values of particle size index.

## 1. Introduction

Wheat grain quality depends on many traits, among which the most important include, e.g., protein content, composition of high-molecular glutenin subunits and grain hardness. In breeding, selection of high-quality wheat is based mainly on the Zeleny sedimentation test, the falling number and the protein content. In most European countries as well as in Canada and the USA grain hardness is also determined during the early stages of breeding.

Grain hardness is not a clearly defined parameter. It may generally be formulated as a resistance to plastic strain and cracking at a force concentrated on the surface of a given body. Several techniques are employed to determine grain hardness, *i.e.*, static methods, such as measurement of the so-called microhardness adopted to cereal materials [1], as well as dynamic methods, such as measurement of the wheat hardness index (WHI) [2], particle size index (PSI) [3], or pearling resistance index (PRI) [4]. Hardness may also be directly determined with the use of near-infrared technique (NIR) spectroscopy [5,6] or Perten Single-Kernel Characterization System (SKCS) [7]. Different principles underlying the evaluation of hardness as well as the complex structure of individual parts of grain cause a considerable variation in hardness estimation. Thus the degree of association between hardness and specific technological characteristics of wheat grain also varies. However, it is generally accepted that grain hardness has a significant effect on some technological properties, particularly sifting capacity, grist sorting capacity, starch damage during milling, susceptibility to amylolytic enzymes, improved fermentability, water absorption of flour, and improved baking value of produced bread [8–11].

Several studies indicate that grain hardness is controlled by the main hardness locus (*Ha*) located at the short arm of chromosome 5D [12–14] which is closely linked to genes encoding *puroindolines a* and *b* (*Pina*, *Pinb*). Soft-grained wheats have the wild allele of the *Pina* gene and accumulate both puroindolines on the surface on starch granules, whereas medium- and hard-grained wheats have mutated alleles at *Pinb* and have reduced amounts or no *puroindoline b* on starch granules. The effect of puroindolines on dough and breadcrumb properties is ascribed to their affinity to lipids [15–17]. However, it was found that the *H*a locus does not explain all the variations observed in grain hardness of wheat populations [10]. Several quantitative trait loci (QTLs) and microsatellite markers associated with grain texture have been reported, among others, by [12,18–24].

Physical methods of hardness estimation are time-consuming and are rarely used in wheat breeding. The near-infrared (NIR) technique is of greatest interest to breeders, because it is easy, rapid and non-destructive, but it only provides a relative evaluation of hardness [5,10,25,26]. It is important for breeders to know whether the grain hardness evaluated by the NIR technique may be a good predictor of dough and baking properties.

The aim of this study was to evaluate the dependence between grain hardness and dough rheology as well the relationship of baking properties of winter wheat breeding lines with hardness of grain.

## 2. Results and Discussion

### 2.1. Results

#### 2.1.1. Molecular Analysis

The material for the present study covered 24 winter wheat genotypes with significantly varied grain hardness. On the basis of the grain hardness determined by using the NIR technique and particle size index (PSI), the studied genotypes were divided into three groups: Hard-, soft- and medium-grained (Table 1).

The studied wheat genotypes of different grain hardness were subjected to molecular analyses. In our earlier studies [27], puroindoline alleles *Pina-D1a, Pina-D1b, Pinb-D1a and Pinb-D1b* had been identified using primer sets described by Lillemo *et al*. [28] and Li *et al*. [29], respectively. All the lines classified as soft in the present study on the basis of physical (NIR and PSI) methods appeared to have wild-type alleles of *Pina-D1a* and *Pinb-D1a*, whereas hard-grained lines in most cases (7 out of 11) had one mutated allele: *Pinb-D1b* (lines 1, 9, 20, 29, 31, 40) or *Pina-D1b (line 35)*, but four lines were identified to have the same set of alleles as soft-grained (Table 1). Among the four medium-grained groups, two lines with wild-type (both *Pina-D1a* and *Pinb-D1a)* alleles as well as two lines with one mutated allele were detected.

**Figure 1:**
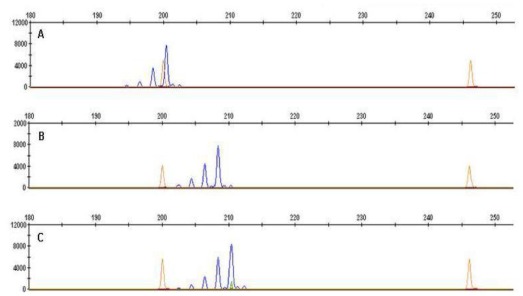
Microsatellite (SSR) products amplified by primer *Xgwm 190* for wheat lines: (**A**) CHD 169/04 (200 bp), (**B**) CHD 760/4 (208 bp) and (**C**) CHD 65/04 (210 bp).

For better characterization of the studied lines, three microsatellite (SSR) markers linked to the *Ha* locus were additionally applied in the present study: *Xgwm190* [18], *Xgwm205* and *Xgwm358* [19]. SSR markers *Xgwm190* (200, 208 and 210 bp), *Xgwm205* (134, 136 and 138 bp), *Xgwm358* (156, 158 and 162 bp) provided the same size of DNA amplified products (200, 136 and 158 bp, respectively) in all soft-grained lines, whereas in hard- and medium-grained groups, the same markers produced different products (Table 1). In Figure 1 SSR products amplified by marker *Xgwm 190* for selected lines are presented.

#### 2.1.2. Variation in Quality Parameters

Mean values of grain hardness and quality parameters assessed using the NIR system, farinograph, mixograph and test baking are presented in Table 2.

Analysis of variance showed a significant variation of breeding lines in terms of all NIR, farinograph and reomixer parameters with an exception for two mixing characteristics: Initial slope (RM3) and peak height (RM8). Among the baking parameters, only bread yield variation was significant. The level of fertilization had a significant effect on most analyzed quality parameters. Exceptions included the following farinograph parameters: Dough consistency, water absorption, dough stability time and reomixer peak height. The genotype-environment (GE) interaction was significant for PSI, most farinograph parameters (except for dough consistency) as well as for four mixing parameters, *i.e.*, area under centre line up to peak (IHTP), time 1–2 (RM4), peak time (RM6) and bandwidth at 10 min (RM11). GE interaction was insignificant for all bread quality parameters (Table 3).

#### 2.1.3. Contrast Analysis

Differences between the mean values of the studied characteristics for hard- and soft-grained lines examined at standard and increased fertilzation levels were estimated and tested by *F* statistics. For the comparison of hard and soft lines, two kinds of contrasts (C1 and C2) were constructed, because composition of hard and soft groups of lines established on the basis of physical and molecular analyses was not the same: In C1, the hard group consisting of 11 lines (nos. 1, 9, 20, 29, 30, 31, 35, 36, 38, 40, 46) was contrasted with the soft group, which consisted of eight lines (nos. 2, 7, 17, 41, 42, 44, 45, 48, 49), whereas in C2 the hard group included nine lines (nos. 1, 8, 9, 19, 20, 29, 31, 35, 40) and the soft group 15 lines (nos. 2, 7, 11, 13, 17, 30, 36, 38, 41, 42, 44, 45, 46, 48, 49) (Table 1).

##### 2.1.3.1. NIR Parameters

At both fertilization levels hard-grained wheat types, in comparison to soft-grained wheat types (C1contrast), were characterized by a significantly higher protein content, wet gluten, Zeleny sedimentation value, and a significantly lower starch content (Table 4).

Estimates of contrasts between levels FL1 and FL2 for all the sets of lines indicated that a higher level of nitrogen fertilization caused a significant increase in values of NIR parameters defining grain hardness, protein content and wet gluten as well as Zeleny sedimentation value, and a reduction of starch content. Differences between the values of PSI parameters on FL1 and FL2 appeared to be insignificant, whereas NIR values were significantly higher on FL2 (Table 4).

Estimates of C2 contrast showed significant differences between H and S groups of lines at both fertilization levels only in NIR values, whereas in the case of the other characteristics tested, differences were significant only on FL2. Moreover, differences in protein content were insignificant both on FL1 and FL2 (Table 4).

##### 2.1.3.2. Farinograph Parameters

Hard-grained lines had significantly higher values of water absorption and dough stability than soft-grained wheat types at both the fertilization levels. The rest of the farinograph parameters were similar for both groups of lines except for dough softening, which was significantly higher for soft-grained lines at FL1 (Table 4, C1 contrast).

Values of the farinograph parameters defining dough consistency, water absorption and dough stability were similar at both fertilization levels. Dough development time and the farinograph quality number for all the studied lines treated together were higher at FL2, while dough softening was higher at FL1 (Table 4).

Comparison of H and S groups of lines constructed on the basis of molecular data indicated significant differences between these groups in the farinograph quality number on both fertilization levels (insignificant in C1). Different results were obtained for dough stability: An estimate of C1 contrast showed better dough stability for soft-grained lines, whereas C2 showed the same for hard-grained ones. For the rest of farinograph parameters differences estimated in C2 contrast were markedly important only at FL1 or FL2 fertilization levels (Table 4).

##### 2.1.3.3. Reomixer Parameters

The following four reomixer parameters were generally higher at the lower fertilization level: IHTP, RM4, RM6 and RM11. The maximum peak height (RM8) was similar for both fertilization levels and both groups of genotypes.

Hard-grained lines were assessed to have significantly higher values of mixing parameters (IHTP, RM4 and RM6) at both fertilization levels (Table 4, Contrast 1).

Differences between hard- and soft-grained groups of lines tested in C2 contrast were found to be significant only for IHTP and RM6 parameters on FL1.

##### 2.1.3.4. Bread-making Properties

The bread yield—determined as bread weight produced from 100 g of flour—was significantly higher in the case of grain produced at FL1 than FL2. Test baking also showed that a greater loaf volume was obtained from grain produced at the lower fertilization level. An increase in the applied nitrogen dose also caused a significant increase in values of breadcrumb grain.

Estimates of C1 contrast showed that a higher bread yield was obtained from hard-grained wheat lines than that from soft-grained ones at both fertilization levels. This was related to higher protein and gluten contents in hard grain. Differences between hard- and soft-grained lines with respect to loaf volume and crumb grain were insignificant.

No significant differences in bread-making properties were ascertained between hard- and soft-grained groups tested in contrast C2 (Table 4).

#### 2.1.4. Correlations

Correlation between PSI and NIR was negative and significant at both fertilization levels. Correlation coefficients between the grain hardness determined by both methods at two levels of nitrogen fertilization and the studied characteristics were significant for sedimentation value, bread yield, and the two mixing parameters IHTP and RM11. The association between grain hardness and other technological parameters was frequently important only at one fertilization level. At lower fertilization levels, the following association between PSI values and farinograph parameters were found: Water absorption, dough stability and dough softening. Whereas at increased levels the following correlations were found: Between protein and starch content, dough consistency and water absorption, and the two mixing parameters RM4 and RM6 (Table 5).

Hardness determined by NIR correlated significantly at both levels of fertilization with wet gluten, dough consistency, water absorption, degree of softening, IHTP, RM4, RM6, RM12 and bread yield. Association between NIR values and other bread-making parameters was insignificant except for breadcrump grain at FL2. It can be seen from Table 5 that the association between NIR parameters and technological properties was more frequently significant than with PSI parameters.

No significant dependencies were observed between both PSI and NIR parameters and the two farinograph parameters dough development time and quality number, and the two mixing parameters RM3 and RM8, and loaf volume.

### 2.2. Discussion

In this study, the relationships between grain hardness and some quality parameters were studied in winter wheat breeding lines examined under field conditions at two levels of nitrogen fertilization. Grain hardness was determined by the PSI and NIR spectroscopy; values of these two parameters were negatively significantly correlated (*r* = −0.601 and *r* = 0.578 at FL1 and FL2, respectively, *p* < 0.01), similar to values observed in our earlier studies [27]. High values of NIR indicate hard grain, whereas higher values of PSI indicate soft grain. It was found that the higher levels of nitrogen fertilization resulted in an increased grain hardness. Moreover, wheat genotypes classified as hard, at both levels of nitrogen fertilization had higher a protein content, wet gluten and Zeleny sedimentation values than those classified as soft.

Quality traits studied were influenced both by genotype and environment with an exception for three characteristics: Initial slope, loaf volume and breadcrumb grain, which appeared to be influenced only by environment. Interaction of genotype × environment (nitrogen fertilization level in this case) was significant for the grain hardness evaluated by PSI parameter and for some farinograph and mixograph characteristics. Similar results were reported by Tsilo and co-workers [24] who recorded a GE interaction only for two mixograph parameters and no GE interaction for all studied bread-making properties.

In the present study, grain hardness was evaluated by physical methods supplemented by molecular analysis, which permitted a better characterization of the studied materials. The results of molecular analyses were in agreement with those obtained by the use of physical methods only in the case of soft-grained lines. Some lines classified by physical methods as hard appeared to have the wild-type *Pina* and *Pinb*, alleles, similar to soft lines. Lines with no clearly determined grain hardness had wild-type or mutated alleles of *Pin*-genes. Moreover, differences in dough and bread-making properties between lines classified as hard and soft on the basis of molecular data appeared to be not so clear as the differences between lines classified as hard and soft on the basis of physical analyses of grain texture. This indicates that the hardness was controlled not only by *Ha* locus, but also by other regions of the genome in the studied grain material. Similar observations have been reported in several other studies [10,12,19–24]. This finding also suggests that selection of wheat for good quality based only on molecular markers connected with *Ha* locus may be ineffective.

In our study, the relationship between grain hardness and other quality parameters were evaluated. Correlation coefficients between grain hardness determined both by PSI and NIR methods and studied traits at both levels of nitrogen fertilization were significant for the following sedimentation value and mixing parameters: IHTP and bandwidth at 10 min. Among the parameters describing baking quality, grain hardness appeared to be significantly correlated only with bread yield. No association was found between grain hardness and other final baking quality parameters, such as loaf volume or crumb grain. This may be a result of the influence of other components of grains, which were not determined in this study. Hruskova [30] reported a positive correlation between grain hardness determined by NIR technique and Zeleny sedimentation value, protein content and 1000-grain weight. In hard red wheat, Tsilo and co-workers [24] found a significantly positive dependence between endosperm texture, evaluated using single kernel characterization system, and some mixograph parameters, but correlation between endosperm texture and bread-making characteristics appeared to be insignificant.

In the present study, values of grain hardness measured by NIR technique correlated more frequently with dough properties than those determined by PSI. In addition, correlation coefficients between NIR, farinograph and reomixer parameters were significant at both fertilization levels, whereas with PSI, this was more frequently found at only one level—FL1 or FL2. The association between grain hardness and protein content was not clear; NIR values correlated significantly with protein content (*r* = 0.445, *p* < 0.01) at the lower fertilization level, whereas PSI values were significantly associated with protein content (*r* = −0.288, *p* < 0.05) only at the increased nitrogen fertilization level. This suggests that these two traits may be independent, as shown previously by Bushuk [31]. It was found that QTLs associated with grain hardness of wheat are located on different chromosomes: 1A, 2A, 5A, 7A, 2B, 6B, 2D, 5D and 6D [12,20–21,24]. Similarly, several QTLs involved in the regulation of PC, not linked to QTLs for grain hardness, were found on different chromosomes, for example 2D [32], 6A, 1B, 6D [23], 1B and 6B [24]. In contrast, significant a association between PC and grain hardness (evaluated by PSI) was recorded by Moiraghi *et al*. [33]. Galande and co-workers [20] found three markers associated with both traits, *i.e.*, grain hardness and PC, in a population of inbred lines derived from a cross between hard and soft wheat genotypes, which may indicate that loci contributing to these traits are linked to each other. Tsilo *et al.* [24] also found QTL on the 5A chromosome that influenced both grain texture and PC. In our study, the analyzed wheat genotypes were of a different pedigree and, as a consequence, of a diverse genetic background, thus these two traits might be controlled by different regions of the genome in these particular breeding lines. This is probably the reason for which the relation between grain hardness and protein content was not as clear as in a population of lines with the same pedigree.

Our results indicate that grain hardness determined by NIR technique may be used in breeding programs as a predictor of dough rheology and baking quality, although at a limited scale. On the other hand, evaluation of grain hardness by NIR technology is a quick and non-destructive method and can be used for a mass selection at the early stage of wheat breeding, e.g., from the F3 generation, *i.e.*, when grain samples are big enough for analyses. In successive generations, the evaluation of grain hardness may supply information on the composition of high molecular weight glutenin subunits, which is being used for the selection of genotypes with improved dough properties. Rheological and baking tests are time-consuming and are often performed at the final stages of cultivar development.

## 3. Experimental Section

### 3.1. Plant Materials

The material for this study comprised 23 advanced breeding lines of winter wheat and a standard *cv*. Tonacja (Table 1). These lines were selected from among 50 lines analyzed in a previous experiment [26], in which grain hardness was evaluated using PSI and torque technique.

The field experiment was conducted in the season of 2008/2009 on an experimental plot in Choryń near Leszno (Poland, the Wielkopolska region) on light brown soil using two fertilization levels: Standard (FL1) at 69 kg·ha^−1^ P, 120 kg·ha^−1^ K and 147 kg·ha^−1^ N, and increased (FL2) at 69 kg·ha^−1^ P, 120 kg·ha^−1^ K and 187 kg·ha^−1^ N. The experiment was established in a complete blocks design in two replications on plots of 5 m^2^, with a row spacing of 12.5 cm. Seeds were sown on 29 September 2008, while harvest was performed on 28 July 2009.

### 3.2. Grain Hardness Determination

Grain hardness was determined using two methods: (1) By measurement PSI [3] with the use of a Quadrumat-Junior mill and a ZBPP flour sifting machine and (2) by using a NIR System Infratec 1241 Analyzer (Foss, Hillerod, Denmark) fitted with a sample transport module and standard sample cups. Samples were scanned from 570 to 1050 nm, and data were collected at every 2 nm. The calibration was supplied by the equipment manufacturer.

The NIR technique was also used to determine protein content, starch content, wet gluten and Zeleny sedimentation value.

### 3.3. Molecular Analysis

DNA extraction: Leaves of 14-day-old wheat plants were used for genomic DNA extraction (Promega Kit). The extracts were diluted to 100 ng/mL and stored at −20 °C.

Puroindoline identification in the studied lines had been performed earlier [27]. In the present study, three microsatellite markers closely linked to the *Ha* locus were used for better characterization of the studied wheat lines: *Xgwm190* [18], *Xgwm205* and *Xgwm358* [19]. For PCR amplification, the Applied Biosystem thermal cycler was used. PCR reaction was performed in 25 μL volumes containing 250 nM of each primer, 0.2 mM of each of dNTP, 1× PCR buffer, 1.5 mM of MgCl_2_, 1.0 unit of Taq DNA polymerase, and 50 ng of genomic DNA. The samples, denatured at 94 °C, were submitted to 45 cycles of 1 min denaturation at 94 °C, 1 min annealing at 55–60 °C (depending on primer *T*_m_), and 2 min elongation at 72 °C, with a final extension of 10 min at 72 °C at the end. Microsatellite alleles were detected on the 3130 Genetic Analyzer (Applied Biosystems, Life Technologies, Grand Island, NY, USA).

### 3.4. Rheological Analysis

The rheological properties of dough were analyzed on a microscale using farinograph and Reomixer. Ten g flour samples of 14% moisture were analysed.

Farinograph analysis was made using a type E farinograph (Brabender, Duisburg, Germany) in a Mixer S10. Five rheological parameters were recorded: Consistency, water absorption, dough development time, dough stability and dough softening at 10 min. The farinograph quality number was recorded as well. Measurements were taken in accordance with a procedure described in the AACC Standard 54–21.02 [34] and ICC 115/1 [35]. During the first measurement, water absorption of flour was determined at 500 Brabander units, following this successive measurement were performed knowing the amount of water required for the proper measurement of farinograph parameters.

**Figure 2:**
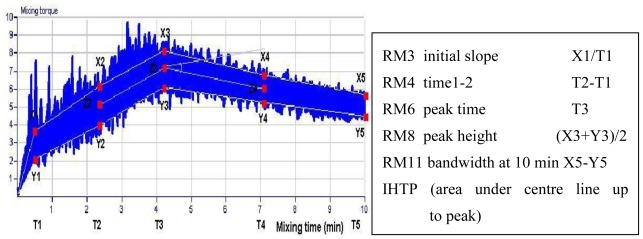
Definition of Reomixer mixing parameters.

Dough mixing was measured using a Bohlen Reomixer (Reologen i Lund AB, Lund, Sweden), which is a planetary pin mixer similar to the Mixograph (National Manufacturing, USA, NE) with a 10 g flour capacity, a mixing speed of 88 rpm and data recording at 10 points/s [36–37]. The process of dough mixing was monitored for 10 min at a temperature of 30 °C. At the end of mixing, 16 pre-selected parameters were automatically extracted from the Reomixer trace. Of these parameters, 5 were selected covering all phases of dough development and describing all basic rheological aspects of mixing characteristic: Area under center line up to peak (IHTP), initial slope (RM3), time 1–2 (RM4), peak time (RM6), peak height (RM8), and bandwidth at 10 min (RM11). The definitions of mixing parameters are presented in Figure 2. Samples were supplemented with 2% NaCl solution at 5.84–6.25 mL depending on the protein content in the sample.

### 3.5. Bread-Making Quality

Baking quality was evaluated on the basis of test baking, in which bread yield in comparison to the flour used (%), loaf volume (cm^3^) and crumb grain number according to the Dallmann scale [38] were assessed. The baking test was performed using three 100 g flour samples corrected to 14% moisture for each line and replicated at FL1 and FL2. Flour samples were mixed with distilled water up to 350 farinograph units, 9 g fresh baking yeast and 3 g salt. Next, dough was placed in a fermentation cabinet at 30 °C and a humidity of 80%. After 60 min, the first dough punching was performed, followed by another such procedure after another 30 min. Subsequently, each piece of dough was molded and placed in the baking pan for 50 min proofing. Thereafter, the dough in pans was baked for 20 min in an oven at 230 °C. Twenty four hours after the completion of the test baking, the above-mentioned baking parameters were evaluated.

### 3.6. Statistical Analysis

The results of the observations and measurements were statistically processed using 2-way analysis of variance. The hypotheses claiming a lack of influence of fertilization level, genotype and interaction of genotype × fertilisation level (GE) were verified. Contrasts between fertilization levels F1 and F2 and between groups of lines with hard and soft grain were estimated and tested by *F* statistic. Two kinds of contrasts were constructed: C1—for the evaluation of differences between hard and soft groups of lines, which were established on the basis of physical measurements, and C2—for the evaluation of differences between groups of lines classified as hard or soft on the basis of molecular markers only. The C1 contrast lines of not clearly defined grain hardness (lines No. 8, 11, 13, 19) were excluded from the analysis.

## 4. Conclusions

The obtained results suggest that selection of wheat for good quality based only on molecular markers connected with the *Ha* locus may not be fully effective. Grain hardness of winter wheat breeding lines was higher at an increased nitrogen fertilization level than at the standard fertilization level. Differences in values of NIR and PSI parameters between hard and soft wheat types were more marked at standard fertilization.

Values of NIR parameter determining grain hardness correlated with quality characteristics at a higher degree than values of particle size index. The NIR correlated with wet gluten and sedimentation value as determined by NIR technique. A significant association was also found between NIR and farinograph parameters such as dough consistency, water absorption capacity and dough stability time, and some reomixer characteristics: Area under center line, time 1–2, peak time and bandwidth at 10 min. A significant relationship was ascertained between NIR and bread yield. This means that grain hardness evaluated by NIR technique may be used as a criterion in breeding selection of winter wheat breeding lines with improved quality.

## Figures and Tables

**Table 1:** Grain hardness, protein content and molecular characterization of studied winter wheat phenotypes.

Line No.	Code	Mean Values at Two Fertilization Levels			Puroindoline Alleles		Microsatellite Markers		

		Protein Content	Hardness NIR	PSI	*Pina* [26]	*Pinb* [26]	*Xgwm 190* (bp)	*Xgwm 205* (bp)	*Xgwm 358* (bp)
Hard-grained lines*									

1	CHD 169/04	13.3	67.9	19.5	*D1a*	*D1b*	200	138	162
9	DED 4152/03	13.3	60.4	20.6	*D1a*	*D1b*	200	138	162
20	LAD 252/04	12.9	62.1	19.4	*D1a*	*D1b*	210	138	162
29	CHD 760/04	14.9	70.7	18.7	*D1a*	*D1b*	208	136	162
30	CHD 65/04	15.6	61.4	19.2	*D1a*	*D1a*	210	138	156
31	CHD 114/04	13.6	62.0	18.6	*D1a*	*D1b*	208	138	156
35	CHD 382/04	14.2	65.2	16.3	*D1b*	*D1a*	210	138	156
36	CHD 565/04	13.7	69.1	20.1	*D1a*	*D1a*	208	134	156
38	CHD 737/04	14.2	65.6	18.7	*D1a*	*D1a*	208	138	162
40	DED 3481/03	14.6	71.8	18.1	*D1a*	*D1b*	208	138	162
46	LAD 319/04	13.9	61.3	20.8	*D1a*	*D1a*	210	138	162

Soft-grained lines*									

2	CHD 329/04	13.7	53.2	22.0	*D1a*	*D1a*	200	136	158
7	DED 5854/03	14.6	51.8	21.4	*D1a*	*D1a*	200	136	158
17	LAD 180/05	13.4	48.7	22.0	*D1a*	*D1a*	200	136	158
41	DED 5845/03	14.0	44.4	24.9	*D1a*	*D1a*	200	136	158
42	DED 5897/03	13.8	43.8	21.2	*D1a*	*D1a*	200	136	158
44	DED 6986/03	13.3	40.9	24.2	*D1a*	*D1a*	200	136	158
45	LAD 125/04	13.6	40.6	31.5	*D1a*	*D1a*	200	136	158
48	LAD 355/04	12.7	48.2	23.1	*D1a*	*D1a*	200	136	158
49	TONACJA	13.2	53.6	21.3	*D1a*	*D1a*	200	136	158

Medium-grained lines*									

8	DED 6240/03	13.5	58.9	21.3	*D1a*	*D1b*	200	136	158
11	DED 6658/03	13.8	54.5	22.5	*D1a*	*D1a*	208	138	162
13	LAD 166/05	13.2	55.7	22.2	*D1a*	*D1a*	208	136	162
19	LAD 245/04	13.7	58.6	21.0	*D1a*	*D1b*	210	138	162

* Hard-grained: near-infrared (NIR) > 60 and particle size index (PSI) < 21; soft-grained: NIR < 54 and PSI > 21; medium-grained: 60 > NIR < 54.

**Table 2:** Mean values and ranges for grain hardness and wheat quality characteristics of wheat lines assessed at two fertilization levels (**FL1**, **FL2**).

Character	FL1		FL2	
	
	Mean	Range	Mean	Range
Grain hardness—PSI	20.7	15.4–30.3	21.5	14.0–33.0
Grain hardness—NIR	53.1	24.7–71.2	61.4	46.5–74.5
Protein content (%)	12.8	11.4–14.5	14.8	13.6–16.7
Starch content (%)	67.3	64.2–69.7	64.8	62.4–66.9
Wet gluten (%)	26.6	24.6–31.7	32.2	29.6–36.5
Zeleny sedimentation value (mL)	37.7	25.3–53.8	55.0	42.2–64.7

**Farinograph Parameters**				

Consistency (FU)	504.6	492.0–520.5	501.8	486.0–520.0
Water absorption (%)	70.0	66.3–74.0	70.4	64.4–73.6
Development time (min)	2.25	1.45–3.05	2.46	1.55–3.30
Dough stability (min)	3.24	2.00–5.00	3.23	1.95–5.30
Degree of softening (FU)	92.5	66.5–133.0	85.8	62.5–114.0
Farinograph quality number	35.2	20.0–49.0	38.0	21.0–60.0

**Dough Mixing Parameters**				

Area under line (IHTP)	23.6	13.0–42.5	20.4	11.5–44.2
Initial slope (RM3)	4.04	3.20–5.22	5.37	4.52–6.04
Time 1–2 (RM4) (min)	2.67	1.17–4.53	2.06	1.21–3.92
Peak time (RM6) (min)	5.86	3.76–8.55	4.10	3.03–5.83
Peak height (RM8)	5.93	5.37–7.05	5.82	5.04–7.67
Bandwidth at 10 min (RM11)	2.20	1.95–2.49	2.08	1.92–2.36

**Bread-Making Parameters**				

Bread yield (%)	140.5	135.2–145.8	142.1	138.4–146.4
Loaf volume (cm^3^)	634.0	586.0–687.0	615.8	557.0–652.0
Bread crumb grain	5.35	3.0–8.0	6.75	5.0–9.0

**Table 3:** *F* statistic values from two-way analysis of variance for grain hardness and quality characters of wheat lines assessed at two fertilization levels.

	Source of Variation		
	
Character	Genotype (G); d.f. = 23	Fertilisation Level (E); d.f. =1	Interaction GE; d.f. = 23
Grain hardness—PSI	20.79 **	9.40 **	3.39 *
Grain hardness—NIR	16.77 **	79.23 **	1.27
Protein content (%)	6.02 **	32.43 **	1.24
Starch content (%)	11.65 **	32.83 **	1.03
Wet gluten (%)	5.43 **	32.70 **	1.19
Zeleny sedimentation value (mL)	8.22 **	50.01 **	1.32

**Farinograph Parameters**			

Consistency (FU)	1.77 *	2.65	0.66
Water absorption (%)	10.34 **	2.63	4.58 **
Development time (min)	11.98 **	20.48 **	2.82 **
Dough stability (min)	53.11 **	0.06	7.10 **
Degree of softening (FU)	17.00 **	20.33 **	2.70 **
Farinograf quality number	22.25 **	18.93 **	5.93 **

**Dough Mixing Parameters**			

Area under line (IHTP)	83.13 **	67.02 **	10.13 **
Initial slope (RM3)	0.57	49.73 **	0.41
Time 1–2 (RM4) (min)	45.96 **	162.83 **	6.23 **
Peak time (RM6) (min)	13.89 **	153.71 **	4.02 **
Peak height (RM8)	0.60	0.21	0.23
Bandwidth at 10 min (RM11)	8.91 **	31.72 **	0.95 *

**Bread-Making Parameters**			

Bread yield (%)	2.91 **	16.28 **	1.09
Loaf volume(cm^3^)	1.57	17.13 **	1.00
Bread crumb grain	0.015	0.470 **	0.092

* *p* < 0.05;

** *p* < 0.01.

**Table 4:** Estimates and results of testing contrasts between hard- and soft-grained wheat lines for quality characteristics at two fertilization levels (FL1, FL2).

Character	Contrast: FL1–FL2	Contrast 1: Hard–soft		Contrast 2: Hart–soft	
	
		FL1	FL2	FL1	FL2
Grain hardness—PSI	−0.84 *	−3.29 **	−3.01 **	−1.56	−2.53 *
Grain hardness—NIR	−8.28 **	18.82 **	11.18 **	8.01 **	6.07 *
Protein content (%)	−2.03 **	0.42 **	0.41 *	−0.03	0.37
Starch content (%)	2.51 **	−1.28 **	−1.10 **	−0.34	−0.59
Wet gluten (%)	−5.55 **	1.29 **	1.04 *	−0.13	1.02 *
Zeleny sedimentation value (mL)	−17.29 **	6.99 **	4.76 **	1.57	3.48 *

**Farinograph parameters**					

Consistency (FU)	2.71	3.01	3.91	4.24	4.21
Water absorption (%)	−0.40	4.00 **	1.84 **	1.45 *	−0.11
Development time (min)	−0.21 **	0.05	−0.03	0.34 **	0.02
Dough stability (min)	0.01	0.42 **	0.55 **	−0.40 **	−0.26 *
Degree of softening (FU)	6.67 **	−11.62 **	−4.02	−4.33	−0.93
Farinograph quality number	−2.81	** 0.33	−0.22	2.42 **	−4.43 **

**Dough mixing parameters**					

Area under centre line (IHTP)	3.28 **	12.47 **	7.62 **	2.94 *	0.16
Initial slope (RM3)	−1.32 **	−0.31	−0.28	−0.32	0.05
Time 1–2 (RM4) (min)	0.61 **	1.11 **	0.63 **	0.19	−0.09
Peak time (RM6) (min)	1.76 **	2.26 **	0.74 **	0.34 *	0.06
Peak height (RM8)	0.12	0.21	−0.20	0.13	0.12
Bandwidth at 10 min (RM11)	0.11 **	0.19 **	0.16 **	0.07	0.02

**Bread-making parameters**					

Bread yield (%)	−1.55 **	2.20 **	1.64 **	0.95	1.81
Loaf volume(cm^3^)	18.27 **	−1.06	3.92	4.66	−2.17
Bread crumb grain	−1.39 **	0.44	−0.31	0.02	−0.20

* *p* < 0.05;

** *p* < 0.01.

**Table 5:** Correlation coefficients between grain hardness measured by PSI and NIR technique (NIR) and quality characters of wheat lines assessed at two fertilization levels (FL1, FL2).

Character	Grain Hardness—PSI		Grain Hardness—NIR	
	
	FL1	FL2	FL1	FL2
Grain hardness—NIR	−0.601 **	−0.578 **	–	–
Protein content (%)	−0.239	−0.288 *	0.445 **	0.232
Starch content (%)	0.385 **	−0.578 **	−0.550 **	−0.200
Wet gluten (%)	−0.270	−0.182	0.527 **	0.342 *
Zeleny sedimentation value (mL)	−0.446 **	−0.310 *	0.642 **	0.477 **

**Farinograph Parameters**				

Consistency (FU)	−0.159	−0.323 *	0.446 **	0.279 *
Water absorption (%)	−0.597 **	−0.022	0.834 **	0.289 *
Development time (min)	−0.106	0.141	0.085	0.064
Dough stability (min)	−0.284 *	−0.120	0.280 *	0.334 *
Degree of softening (FU)	0.523 **	0.018	−0.427 **	−0.183
Farinograph quality number	−0.051	0.215	0.007	−0.054

**Dough Mixing Parameters**				

Area under line (IHTP)	−0.322 *	−0.349 *	0.616 **	0.535 **
Initial slope (RM3)	0.216	0.023	−0.154	−0.142
Time 1–2 (RM4) (min)	−0.254	−0.341 *	0.516 **	0.485 **
Peak time (RM6) (min)	−0.249	−0.296 *	0.510 **	0.476 **
Peak height (RM8)	−0.223	−0.147	0.261	−0.088
Bandwidth at 10 min (RM11)	−0.446 **	−0.383 **	0.650 **	0.574 **

**Bread-Making Parameters**				

Bread yield (%)	−0.281 *	−0.379 **	0.429 **	0.328 *
Loaf volume (cm^3^)	−0.142	−0.165	0.134	0.051
Bread crumb grain	0.025	−0.022	0.040	0.300 *

* *p* < 0.05;

** *p* < 0.01.
